# Outbreak of Neisseria meningitidis, Edmonton, Alberta, Canada

**DOI:** 10.3201/eid0805.010337

**Published:** 2002-05

**Authors:** Gregory J. Tyrrell, Linda Chui, Marcia Johnson, Nicholas Chang, Robert P. Rennie, James A. Talbot, The Edmonton Meningococcal Study Group

**Affiliations:** * The Provincial Laboratory of Public Health for Alberta, Alberta, Canada; † The University of Alberta, Edmonton, Alberta, Canada; ‡ The Capital Health Authority, Edmonton, Alberta, Canada

**Keywords:** Neisseria meningitidis, outbreak, serogroup C, pulsed-field gel electrophoresis, incidence, Canada

## Abstract

From December 1999 to April 2001, the greater Edmonton region had 61 cases of invasive meningococcal infection, two fatal. The outbreak was due to Neisseria meningitidis serogroup C, electrophoretic type 15, serotype 2a. Analysis of the strains showed that 50 of 56 culture-confirmed cases were due to a single clone and close relatives of this clone. This strain had not been previously identified in the province of Alberta dating back to January 1997

Neisseria meningitidis causes outbreaks of disease resulting in severe illness and death. These outbreaks occur in persons in their teens and early twenties; however, in some outbreaks, the very young (<2 years of age) are also severely affected (1). Persons >25 years of age appear to be less affected. North American outbreaks are confined primarily to serogroup C strains and less commonly to Y and W135 (2–5).

We report an outbreak of a serogroup C clone of N. meningitidis in the Edmonton region of Alberta, Canada; the serogroup had a unique restriction fragment length polymorphism (RFLP) pattern as determined by pulsed-field gel electrophoresis (PFGE).

## The Outbreak

**Figure 1:**
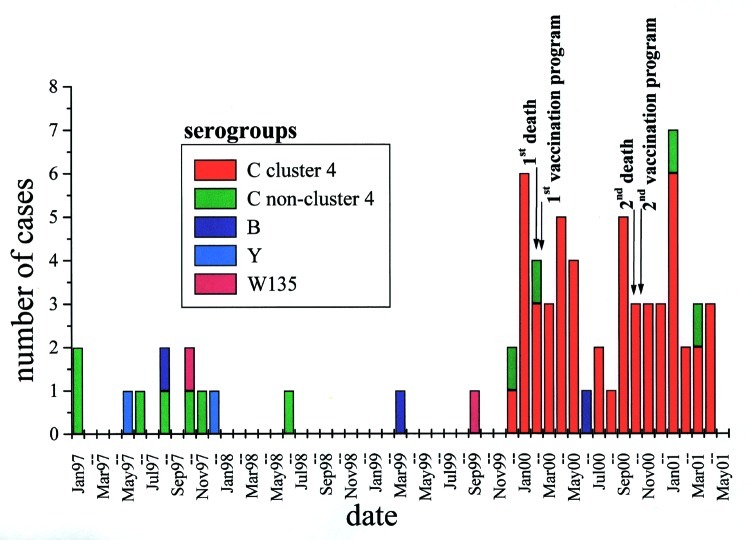
Neisseria meningitidis cases, Edmonton, Alberta, January 1997 to May 2001.* *Cluster 4 refers to clusters derived from restriction fragment length polymorphism patterns, designated in Figure 2.

The Edmonton region has a mixed metropolitan and rural population totaling 827,507 (6). From January 1997 to November 1999 (35 months), this region had 13 cases of culture-confirmed invasive N. meningitidis disease (5 from blood, 6 from cerebrospinal fluid [CSF], and 2 from joints) (Figure 1). Serogroup determination, by the antiserum agar method previously described, showed that these included two cases of serogroup B, seven of serogroup C, two of serogroup W135, and two of serogroup Y (7,8). During this period, the incidence of culture-confirmed meningococcal disease did not exceed two cases per month (Figure 1). However, from December 1999 to April 2001, 61 cases of invasive N. meningitidis disease occurred; 57 of these were confirmed by culture and 4 on the basis of clinical findings, positive results from an antigen detection assay, or both. The culture-confirmed cases were from blood (51 cases) and CSF (6 cases). Of the 57 culture-confirmed cases, 56 were serogroup C, and 1 was serogroup B (blood isolate).

In relation to clinical outcome, 43 (70.5%) of the 61 patients fully recovered; 2 (3.3%) died (a 16-year-old woman and a 19-year-old man, both infected with serogroup C) (Figure 1); 4 (6.6%) required amputations; 7 (11.5%) had severe scars; and 9 (14.8%) had other sequelae such as knee pain, neurologic sequelae, decreased hearing, decreased sensation at the extremities, and stiffness in hands. The ages affected during the outbreak period ranged from 5 weeks to 77 years. Outbreak-associated patients were primarily <24 years of age. Age breakdown showed that 10 (17.9%) of 56 confirmed serogroup C strains were in the birth- to 1-year age group (Table). The conjugate vaccine for use in this age group was licensed in Canada in May 2001 and was therefore not available during the outbreak. The high number of cases in this age group translates into an incidence rate of 50 per 100,000 (Table). In comparison, the most recently published national data show the rate for the group <1 year of age to be 12.9/100,000 for 1997 and 6.5/100,000 for 1998 (9). Also, age groups 15-19 and 20-24 showed unusually high incidences of disease in this outbreak (Table). Thirty patients with culture-confirmed disease were female, and 27 were male. Patients were scattered geographically throughout the region, with no more than one case a close contact of another. All contacts of patients were treated with rifampin. Except for age group, no particular populations were determined to be at greater risk for infection than other.

**Table:** Rates of Neisseria meningitidis disease in the Edmonton, Canada, region (per 100,000)a

Rate	Age (cases)								
	<1	2-4	5-9	10-14	15-19	20-24	25-34	35-59	>60
17-month periodb	50.0 (9)	9.7 (3)	5.3 (3)	6.8 (4)	35.7 (20)	10.6 (6)	4.0 (5)	2.3 (7)	1.7 (3)
Annualized	37.5	7.3	4.0	5.1	26.8	8.0	3.0	1.8	1.3

a Population=827,507 (6). b December 1999 through April 2001.

A vaccination campaign that targeted persons ages 2 to 19 was undertaken in the region from February 14 to 28, 2000, using polysaccharide quadravalent meningococcal vaccine; 168,000 children were immunized. Because of a continuing higher-than-expected number of cases, the vaccine was again offered in October 2000 (Figure 1). This time, the vaccine was offered to all previously unimmunized 2- to 24-year-olds (61,900 doses delivered in 6 days). In April 2001, vaccine was again offered to those 2-year-olds not previously eligible in October 2000. Overall, 87% of people in the targeted age group were vaccinated. After the vaccine campaigns, nine cases of invasive meningococcal disease occurred in those eligible for immunization but not immunized (total population of 2- to 24-year-olds 265,300). Nine cases also occurred in the immunized population, for a calculated vaccine effectiveness of 84%.

## Conclusions

Electrophoretic typing, serotyping, and serosubtyping performed by the National Microbiology Laboratory, Population and Public Health Branch of Health Canada, Winnipeg, Canada, showed that all serogroup C strains belonged to electrophoretic type (ET)15, serotype 2a (10). ET15 entered the Canadian population as early as 1986 in Ontario and has since been demonstrated to be responsible for a number of outbreaks in this country (9,11). The most recent data for ETs in Canada date to 1997 and 1998 (9). During 1997, ET15 accounted for 83.1% of strains analyzed. This proportion increased in 1998 to 93.7%, indicating that ET is the predominant type causing invasive disease in Canada. Serosubtyping showed variation in the class 1 outer membrane protein (OMP1). Strains were either P1.2,5 (27 isolates), P1.2 (24 isolates), P1.15 (2 isolates), or P1.- (3 isolates). The two fatal cases were both P1.2,5.

Serogrouping, ET, and serosubtyping provided accurate characterization of the circulating strains in the Edmonton region; however, they failed to determine if the outbreak was clonal or if the increased cases were due to unrelated N. meningitidis strains. To determine this, RFLP analysis via PFGE was used with minor modifications (12). Bacterial cells were grown on sheep blood agar. The cells were scraped off and placed in 10% formalin for 15 minutes for bacterial inactivation. The cell number was standardized to an optical density of 1.0 at A610. Lysozyme was added to 100 µL of cell suspension at a final concentration of 0.2 mg/mL, mixed gently, and added to 1 mL of 1.6% (W/V) low-melting agarose. The mixture was then transferred to the gel plug mold. The gel plug was suspended in Lysis II solution (1 mg/mL lysozyme, 0.5% Brij 35, 0.2% sodium deoxycholate, and 0.5% sodium lauryl sarcosine in Tris-EDTA buffer) for 1 hour, replaced with ESP buffer (0.5 M EDTA, pH 8.0, 1% sodium lauryl sarcosine, 0.5 mg/mL Proteinase K), and incubated at 50°C for 2 hours. Slices of plug (1X5 mm) were digested by using 30 U of the restriction endonuclease enzyme Spe I (GIBCO BRL, Burlington, Ontario, Canada) for 1.5 hours. The restricted DNA was resolved by PFGE with the following running conditions: initial switch time: 5.0 seconds, final switch time: 25 seconds at 6 V/cm with included angle at 120°C for 20 hours. The gel was stained with ethidium bromide. Analysis was performed by using the BioRad Gel Doc System (Bio-Rad Laboratories, Mississauga, Ontario) and Molecular Analyst Software (Bio-Rad). RFLP pattern numbers were assigned to each isolate with one band difference in the RFLP profile.

**Figure 2:**
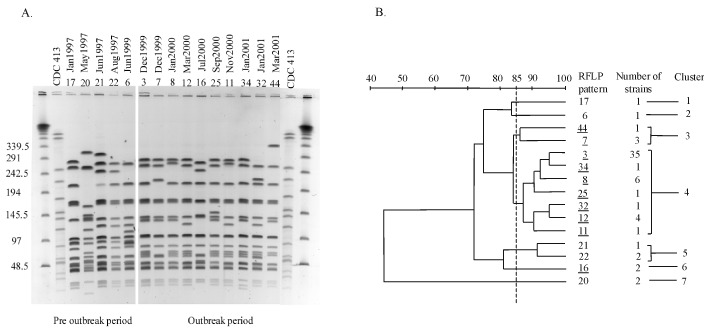
A, restriction fragment length polymorphism (RFLP) analysis of Neisseria meningitidis serogroup C strains generated by pulsed-field gel electrophoresis (PFGE) by using the Spe I restriction endonuclease. The strain CDC-413 was used as a control for the PFGE. B, dendogram analysis generated from “A.” Percent identity is shown at the top. The RFLP pattern designation is shown on the right. RFLP patterns not underlined were seen from January 1997 to November 1999. Underlined RFLP patterns were seen from December 1999 to April 2001. The dashed line indicates 85% identity. A 1% tolerance was used to generate the dendogram.

RFLP analysis showed 15 distinguishable patterns (Figure 2A). Five of these RFLP patterns were seen only from January 1997 to November 1999 (patterns 6, 17, 20, 21, and 22). These patterns were identified by retrospectively determining their RFLP profile from archived strains. The remaining 10 were present only from December 1999 to April 2001 (patterns 3, 7, 8, 11, 12, 16, 25, 32, 34, and 44). Figure 2B shows a dendrogram analysis of the 15 Spe I-generated RFLP profiles generated by using a 1% tolerance. We used an 85% breakpoint to determine relatedness, as reported by Popovic et al. (13). At the 85% relatedness breakpoint, the RFLP profiles formed seven clusters. RFLP patterns for the largest cluster (49 isolates-cluster 4) were only seen during the outbreak period (December 1999 to April 2001). Both deaths were associated with RFLP pattern 3 strains. Interestingly, pattern 7 in cluster 3 was similar on visual examination to pattern 3 (Figure 2A). The first RFLP pattern (first case) detected was pattern 3 (December 24, 1999) followed by pattern 7 (second case, December 29, 1999). Pattern 7 was not detected before December 1999. These data suggest that patterns 3 and 7 arose concomitantly. Even though these patterns appear close in time, pattern 3 and its relatives resulted in 51 cases, whereas pattern 7 was isolated from only 3 cases. Whether pattern 3 strains are more virulent than pattern 7 strains remains to be determined. We have also received reports that this clone has caused disease in other regions of the province of Alberta and in one other Western Canadian province in the same period reported for our outbreak.

In conclusion, the Edmonton region in the province of Alberta, Canada, had an outbreak of N. meningitidis caused by a clone unique to this region. This clone was associated with increased deaths and can readily spread beyond defined geographic boundaries. Other provincial and state laboratories need to be able to recognize this clone should it appear in their area.
